# Determinants of angle kappa in cataract patients

**DOI:** 10.3389/fmed.2025.1726626

**Published:** 2026-01-06

**Authors:** Ling Jin, Xiaoning Hao, Baoyi Liu, Wenwen Geng, Ruirui Ma, Yuling Niu, Duanrong Cao, Yijun Hu

**Affiliations:** 1 Department of Ophthalmology, The People’s Hospital of Baoan Shenzhen, Shenzhen, China; 2 Department of Ophthalmology, The Second Affiliated Hospital of Shenzhen University, Shenzhen, China; 3 Department of Ophthalmology, Zhujiang Hospital, Southern Medical University, Guangzhou, Guangdong, China; 4 Guangdong Eye Institute, Department of Ophthalmology, Guangdong Provincial People’s Hospital (Guangdong Academy of Medical Sciences), Southern Medical University, Guangzhou, China

**Keywords:** angle kappa, biometrics, cataract surgery, determinants, intraocular lens

## Abstract

**Purpose:**

To investigate the determinants of angle kappa in cataract patients.

**Methods:**

In this retrospective study we included biometric data in both eyes of 715 cataract patients measured by Lenstar LS-900. The ocular biometrics included angle kappa (X component, Y component, chord mu), iris center (X component, Y component, iris center distance), central corneal thickness (CCT), corneal curvature (CR), anterior chamber depth (ACD), lens thickness (LT), axial length (AL), anterior corneal astigmatism (ACA), white-to-white corneal diameter (WTW), and pupil size (PS). Correlations of angle kappa with other biometrics were evaluated using Spearman’s correlation tests. Determinants of angle kappa were analyzed using generalized estimating equations.

**Results:**

Chord mu was significantly correlated to ACD, LT, AL, ACA, WTW, PS, iris center X component, and iris center distance (*r* = −0.19 to 0.28, all *P* < 0.05). Determinants of chord mu were ACD (β = −0.05), PS (β = 0.04), and iris center distance (β = 0.17). For the X component of angle kappa, the determinants were ACD (β = 0.10), LT (β = 0.04), AL (β = 0.02), WTW (β = −0.04), PS (β = −0.03), and iris center X component (β = 0.42). For the Y component of angle kappa, the determinants included sex (β = 0.05), PS (β = −0.04), and iris center Y component (β = 0.13).

**Conclusion:**

Different determinants of angle kappa were found in this study. Our findings are important for understanding the influencing biometrics of angle kappa.

## Introduction

Refractive cataract surgery has evolved from mere visual rehabilitation to precise optical reconstruction with widespread adoption of premium intraocular lenses (IOLs) (e.g., toric, multifocal, and extended-depth-of-focus designs) (1, 2). Nevertheless, in some cases significant postoperative visual quality concerns remain, including unsatisfactory visual acuity at specific distances, increased dysphotopsia, reduced contrast sensitivity, and higher intraocular straylight (3). In some cases, IOL exchange may be necessary. However, IOL exchange may be difficult in these cases. Studies have identified blurred vision and photic phenomena, such as glare, as the main complaints from dissatisfied patients, with causes including multifocal IOL decentration, posterior capsular opacification, and uncorrected refractive errors (4, 5). One of the key factors influencing postoperative visual outcomes is the alignment of the IOL, which is affected by the geometry of the eye, particularly by angle kappa (6).

Angle kappa, defined as the spatial offset between the anatomical and visual axes, has been increasingly recognized as an important determinant of premium IOL optical performance (7). Discrepancies in angle kappa can lead to refractive errors and visual disturbances such as astigmatism, glare, and halos (8). Previous studies have highlighted various ocular biometric parameters, such as anterior chamber depth (ACD) and pupil size (PS), that influence angle kappa (9, 10). These parameters contribute to the geometry of the anterior segment, which in turn affects the optical characteristics of the eye. Despite the known importance of angle kappa in cataract surgery, the specific relationship between these ocular biometrics and angle kappa, particularly the X component, Y component, and chord mu, remains under-explored.

The purpose of this study was to evaluate the relationship between ocular biometrics and angle kappa in cataract patients, with a specific focus on the X component, Y component, and chord mu. By using Spearman’s correlation analysis and regression models, we aimed to identify significant determinants of angle kappa and to explore how these factors shape the optical properties of the eye. Our findings will provide valuable insights into the role of ocular geometry in cataract surgery.

## Materials and methods

### Study design and participants

We conducted this cross-sectional retrospective study with adherence to the Declaration of Helsinki and the approval from the Institutional Review Board (IRB) of The People’s Hospital of Baoan Shenzhen (BYL20240638). Written informed consent was waived by the IRB because no participants could be identified from the data.

Patients aged 45 years or older who had been scheduled for cataract surgery and had undergone Lenstar LS-900 examinations with no machine-defined failed data points for any biometric parameters between 2022 and 2024 were included. Examinations of both eyes from each patient were included in the study. The exclusion criteria were a history of ocular surgery or trauma, ocular surface or corneal abnormalities, vitreous or retinal diseases, aphakic and pseudophakic conditions, or any other ocular pathology that might have influenced the accuracy of the Lenstar LS-900 measurements. Ultimately, a total of 1,430 eyes of 715 cataract patients were included in the study.

### Ocular biometrics

**FIGURE 1:**
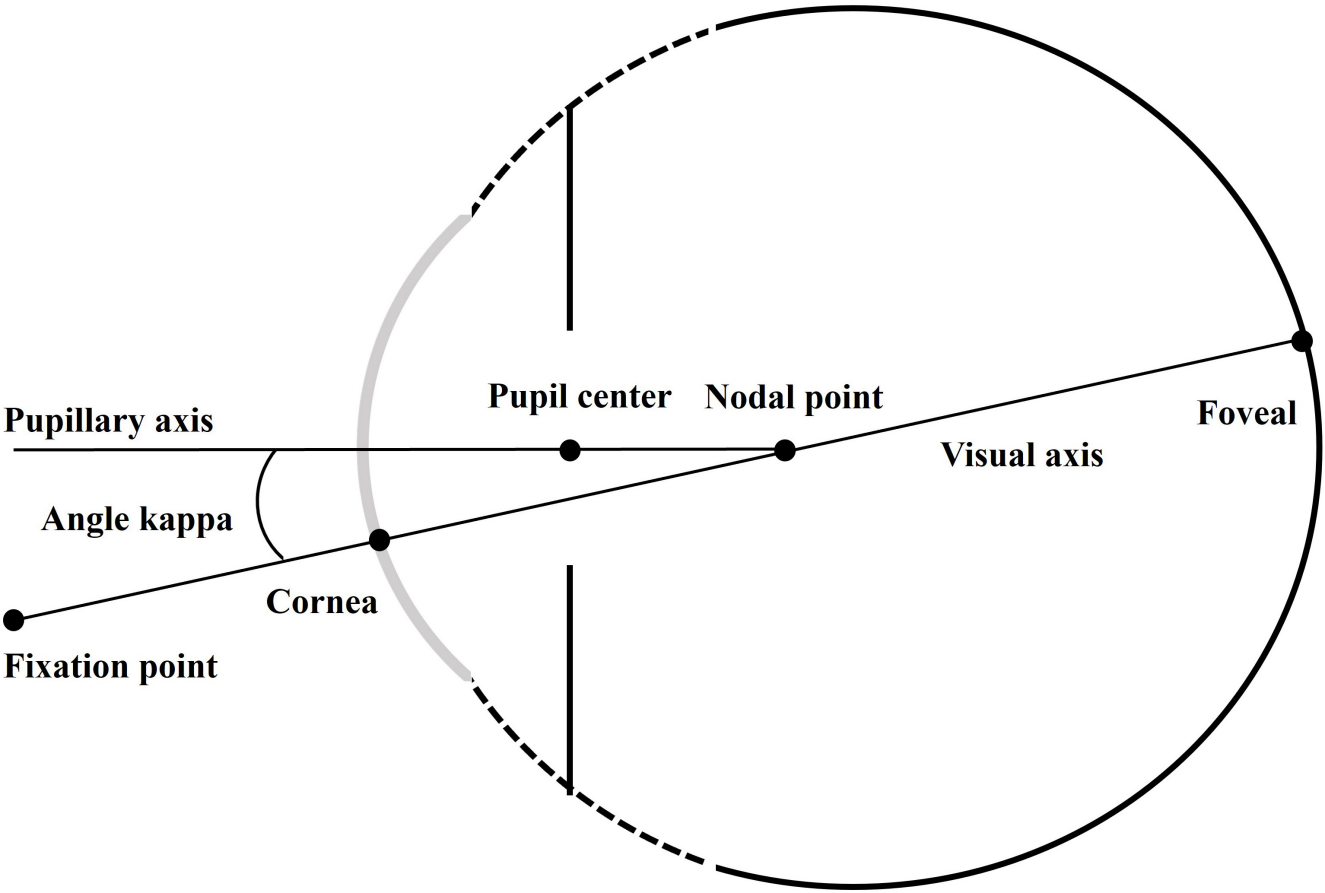
Illustration of angle kappa.

All eyes included in this study underwent a comprehensive preoperative ophthalmic examination, including the evaluation of visual acuity and intraocular pressure, slit-lamp examination, fundoscopy, and Lenstar LS-900 scans. Ocular biometric parameters measured by Lenstar included angle kappa (X component, Y component, chord mu, shown in Figure 1), iris center (X component, Y component, iris center distance), central corneal thickness (CCT), corneal curvature (CR), ACD, lens thickness (LT), axial length (AL), anterior corneal astigmatism (ACA), white-to-white corneal diameter (WTW), and PS. Ocular biometric measurement of the patients were performed by experienced technicians under standard illumination conditions (100–150 lux) at one sitting. At least three consecutive measurements were obtained, and measurements with missing biometric data were excluded. The components of angle kappa are expressed as Cartesian coordinates of pupil center from the corneal light reflex on *X* and *Y* axes. In both eyes, the labels for *Y*-axis component of angle kappa is the same (positive value for upper side and negative value for lower side). The labels for *X*-axis component of angle kappa in both eyes are mirror symmetrical (positive value for nasal side and negative value for temporal side), but this means that the both eyes do not have the same coordinate system. So we have reversed the label for *X*-axis component in the left eye before analysis (positive value for temporal side and negative value for nasal side) (11, 12). Chord mu was calculated using the following formula:


$$\mathrm{Chord}\,\mathrm{mu}=\sqrt{{\left(X-a\,\mathrm{xis}\,\mathrm{component}\right)}^{2}+{\left(Y-\mathrm{axis}\,\mathrm{component}\right)}^{2}}$$

### Statistical analysis

Due to interocular asymmetry of the angle kappa parameters (intra-class coefficients: 0.341 to 0.532), we pooled the right eye and left eye data for analysis. The Shapiro–Wilk test was used to test the normality of variables. Continuous data are presented as mean ± standard deviation, and the corresponding median, quartile, and 95% normal range are also shown. Correlations of angle kappa with other biometrics were evaluated using Spearman’s correlation tests. Determinants of angle kappa were analyzed using univariable and multivariable generalized estimating equations (GEE). The variables found statistically significant in the univariable analysis were entered in the multivariable GEE. Outcomes of the GEE were expressed as β and 95% confidence interval (CI). A *P*-value less than 0.05 was considered statistically significant.

## Results

Of the 715 cataract patients included, 392 (54.8%) were women. The mean age was 65.6 ± 10.2 years. Descriptive statistics for preoperative biometric parameters are presented in Table 1. Angle kappa X component, Y component and chord mu were −0.14 ± 0.21 mm, −0.09 ± 0.20 mm and 0.28 ± 0.18 mm, respectively. Corneal parameters showed CCT of 538.00 ± 34.00 μm, CR of 44.00 ± 1.60 D, ACA of 0.97 ± 0.78 D, and WTW of 12.00 ± 0.59 mm, respectively. Anterior segment parameters revealed ACD of 2.50 ± 0.42 mm, LT of 4.50 ± 0.41 mm, and PS of 4.20 ± 1.40 mm, respectively. AL was 24.00 ± 1.50 mm. Iris center displacements were −0.33 ± 0.23 mm horizontally (X component) and 0.06 ± 0.31 mm vertically (Y component), with linear distance of 0.44 ± 0.25 mm, respectively.

**TABLE 1:** Angle kappa and other biometrics (*n* = 1,430).

Biometrics	Mean ± SD	Median (IQR)	95% range
X component (mm)	−0.14 ± 0.21	−0.14 (0.24)	−0.52, 0.27
Y component (mm)	−0.09 ± 0.20	−0.07 (0.22)	−0.56, 0.24
Chord mu (mm)	0.28 ± 0.18	0.25 (0.22)	0.04, 0.71
CCT (μm)	538.00 ± 34.00	537.00 (42.00)	472.00, 608.00
CR (diopter)	44.00 ± 1.60	44.00 (2.00)	41.15, 47.43
ACD (mm)	2.50 ± 0.42	2.50 (0.60)	1.66, 3.29
LT (mm)	4.50 ± 0.41	4.50 (0.60)	3.68, 5.30
AL (mm)	24.00 ± 1.50	23.00 (1.00)	21.66, 27.91
ACA (diopter)	0.97 ± 0.78	0.79 (0.80)	0.11, 2.86
WTW (mm)	12.00 ± 0.59	12.00 (1.00)	10.15, 12.50
PS (mm)	4.20 ± 1.40	3.70 (1.20)	2.76, 7.99
Iris center X component (mm)	−0.33 ± 0.23	−0.33 (0.23)	−0.85, 0.10
Iris center Y component (mm)	0.06 ± 0.31	0.03 (0.30)	−0.44, 0.75
Iris center distance (mm)	0.44 ± 0.25	0.40 (0.22)	0.11, 1.10

SD, standard deviation; IQR, interquartile range; CCT, central corneal thickness; CR, corneal curvature; ACD, anterior chamber depth; LT, lens thickness; AL, axial length; ACA, anterior corneal astigmatism; WTW, white-to-white corneal diameter; PS, pupil size.

### Correlations of angle kappa with other biometrics

Table 2 quantified the associations between angle kappa X component, Y component and chord mu with key ocular biometric parameters using Spearman’s correlation analysis.

**TABLE 2:** Correlations of angle kappa with other biometrics* (*n* = 1,430).

Biometrics	X component	Y component	Angle kappa
CCT	0.02, (−0.03, 0.07)	−**0.07, (**−**0.12**, −**0.02**), ***P* = 0.008**	0.02, (−0.03, 0.08)
CR	0.03, (−0.03, 0.08)	−0.03, (−0.08, 0.02)	−0.03, (−0.09, 0.02)
ACD	**0.27**, (**0.22**, **0.32**), ***P* < 0.001**	0.01, (−0.04, 0.06)	**−0.17**, (**−0.23**, **−0.12**), ***P* < 0.001**
LT	**−0.13**, (**−0.18**, **−0.07**), ***P* < 0.001**	0.00, (−0.06, 0.05)	**0.07**, (**0.02**, **0.12**), ***P* = 0.010**
AL	**0.23**, (**0.18**, **0.28**), ***P* < 0.001**	0.01, (−0.04, 0.07)	**−0.12**, (**−0.17**, **−0.07**), ***P* < 0.001**
ACA	−0.03, (−0.08, 0.03)	−0.02, (−0.07, 0.04)	**0.06**, (**0.01**, **0.11**), ***P* = 0.025**
WTW	**0.15**, (**0.09**, **0.20**), ***P* < 0.001**	−0.02, (−0.07, 0.04)	**−0.06**, (**−0.11**, **−0.01**), ***P* = 0.023**
PS	**−0.10**, (**−0.15**, **−0.05**), ***P* = 0.001**	**−0.27**, (**−0.31**, **−0.21**), ***P* < 0.001**	**0.28**, (**0.23**, **0.33**), ***P* < 0.001**
Iris center X component	**0.48**, (**0.43**, **0.52**), ***P* < 0.001**	−0.01, (−0.07, 0.04)	**−0.19**, (**−0.24**, **−0.14**), ***P* < 0.001**
Iris center Y component	0.03, (−0.02, 0.09)	**0.26**, (**0.21**, **0.31**), ***P* < 0.001**	−0.05, (−0.10, 0.01)
Iris center distance	**−0.36**, (**−0.41**, **−0.31**), ***P* < 0.001**	0.01, (−0.04, 0.06)	**0.27**, (**0.22**, **0.32**), ***P* < 0.001**

*Presented as Spearman’s correlation coefficients (95% confidence interval) and bold number indicates *P* < 0.05; CCT, central corneal thickness; CR, corneal curvature; ACD, anterior chamber depth; LT, lens thickness; AL, axial length; ACA, anterior corneal astigmatism; WTW, white-to-white corneal diameter; PS, pupil size.

For the X component, iris center X component (*r* = 0.48, 95% CI: 0.43, 0.52) and ACD (*r* = 0.27, 95% CI: 0.22, 0.32) exhibited the strongest positive correlations with angle kappa. This suggests that a deeper ACD and a more left positioned iris center are associated with a left shift of angle kappa in the X direction. Other biometrics such as CCT, CR, and AL showed weak or no significant correlations with the X component, with coefficients ranging from 0.02 to 0.23.

For the Y component, PS demonstrated a significant negative correlation with angle kappa (*r* = −0.27, 95% CI: −0.31, −0.21), suggesting that larger PS is linked to an inferior shift of angle kappa in the Y direction. The iris center Y component also showed a moderate positive correlation (*r* = 0.26, 95% CI: 0.21, 0.31), indicating that a more superior positioned iris is associated with a superior shift in Y component of angle kappa. Other parameters, including CCT, CR, ACD, and AL, had minimal or no significant correlations with the Y component.

For chord mu, iris center distance showed a positive correlation (*r* = 0.27, 95% CI: 0.22, 0.32), indicating that a greater iris center distance corresponds with higher chord mu values. PS showed a significant positive correlation (*r* = 0.28, 95% CI: 0.23, 0.33) with chord mu, suggesting that larger PS is linked to larger chord mu values. ACD exhibited a negative correlation (*r* = −0.17, 95% CI: −0.23, −0.12), indicating a decrease in chord mu with increasing ACD. Other parameters, such as CCT, CR, and AL, showed weak or insignificant correlations with chord mu.

### Determinants of angle kappa

Table 3 presented the determinants of angle kappa across three components: X component, Y component, and chord mu, based on the GEE.

**TABLE 3:** Determinants of angle kappa (*n* = 1,430).

Determinants	Angle kappa X component				Angle kappa Y component				Chord mu			
	Univariable GEE		Multivariable GEE		Univariable GEE		Multivariable GEE		Univariable GEE		Multivariable GEE	
	Beta	95% CI	Beta	95% CI	Beta	95% CI	Beta	95% CI	Beta	95% CI	Beta	95% CI
Age	**−0.003**	(**−0.004**, **−0.002**) ***P* < 0.001**	0.00	(0.00, 0.00)	0.00	(0.00, 0.00)	N/A	N/A	**0.00**	(**0.00**, **0.00**) ***P* = 0.003**	0.00	(0.00, 0.00)
Sex	−0.02	(−0.04, 0.01)	N/A	N/A	**0.06**	(**0.03**, **0.08**) ***P* < 0.001**	**0.04**	(**0.02**, **0.07**) ***P* < 0.001**	−0.01	(−0.03, 0.01)	N/A	N/A
CCT	0.00	(0.00, 0.00)	N/A	N/A	**0.00**	(**0.00**, **0.00**) ***P* = 0.02**	0.00	(0.00, 0.00)	0.00	(0.00, 0.00)	N/A	N/A
CR	0.00	(0.00, 0.00)	N/A	N/A	0.00	(−0.01, 0.01)	N/A	N/A	0.00	(0.00, 0.00)	N/A	N/A
ACD	**0.11**	(**0.08**, **0.14**) ***P* < 0.001**	**0.1**	(**0.06**, **0.14**) ***P* < 0.001**	0.01	(−0.02, 0.04)	N/A	N/A	−**0.05**	**(−0.08**, **−0.03**) ***P* < 0.001**	**−0.05**	(**−0.08**, **−0.02**) ***P* < 0.001**
LT	**−0.04**	(**−0.07**, **−0.02**) ***P* = 0.003**	**0.04**	(**0.00**, **0.07**) ***P* = 0.03**	−0.02	(−0.05, 0.01)	N/A	N/A	0.01	(−0.02, 0.04)	N/A	N/A
AL	**0.04**	(**0.03**, **0.04**) ***P* < 0.001**	**0.02**	(**0.01**, **0.02**) ***P* = 0.001**	0.00	(−0.01, 0.02)	N/A	N/A	−0.01	(−0.02, 0.00)	0.00	(−0.01, 0.01)
ACA	0.00	(−0.02, 0.01)	N/A	N/A	0.00	(−0.01, 0.02)	N/A	N/A	**0.01**	(**0.00**, **0.03**) ***P* = 0.03**	0.01	(−0.01, 0.02)
WTW	**0.04**	(**0.02**, **0.06**) ***P* < 0.001**	**−0.04**	(**−0.06**, **−0.01**) ***P* = 0.01**	−0.01	(−0.03, 0.01)	N/A	N/A	−0.01	(−0.03, 0.01)	N/A	N/A
PS	**−0.02**	(**−0.03**, **−0.01**) ***P* < 0.001**	**−0.03**	(**−0.04**, **−0.02**) ***P* < 0.001**	**−0.04**	(**−0.05**, **−0.03**) ***P* < 0.001**	**−0.04**	(**−0.05**, **−0.03**) ***P* < 0.001**	**0.04**	(**0.03**, **0.05**) ***P* < 0.001**	**0.04**	(**0.03**, **0.05**) ***P* < 0.001**
Iris center X component	**0.41**	(**0.33**, **0.48**) ***P* < 0.001**	**0.42**	(**0.32**, **0.53**) ***P* < 0.001**	0.01	(−0.03, 0.06)	N/A	N/A	−**0.08**	(**−0.14**, **−0.02**) ***P* = 0.02**	0.02	(−0.05, 0.09)
Iris center Y component	0.01	(−0.03, 0.05)	N/A	N/A	**0.13**	(**0.08**, **0.19**) ***P* < 0.001**	**0.13**	(**0.08**, **0.18**) ***P* < 0.001**	−0.01	(−0.05, 0.03)	N/A	N/A
Iris center distance	**−0.21**	(**−0.27**, **−0.16**) ***P* < 0.001**	0.02	(−0.05, 0.10)	0.00	(−0.05, 0.05)	N/A	N/A	**0.18**	(**0.13**, **0.23**) ***P* < 0.001**	**0.17**	(**0.11**, **0.24**) ***P* < 0.001**

GEE, generalized estimating equations; CI, confidence interval; Bold number indicates *P* < 0.05; CCT, central corneal thickness; CR, corneal curvature; ACD, anterior chamber depth; LT, lens thickness; AL, axial length; ACA, anterior corneal astigmatism; WTW, white-to-white corneal diameter; PS, pupil size.

For the determinants of X component in the multivariable model, the iris center X component was the strongest positive determinant, showing a significant positive correlation (β = 0.42, 95% CI: 0.32, 0.53), followed by ACD with a significant positive correlation (β = 0.10, 95% CI: 0.06, 0.14), LT with a significant positive correlation (β = 0.04, 95% CI: 0.003, 0.07), and AL with a significant positive correlation (β = 0.02, 95% CI: 0.01, 0.02). WTW (β = −0.04, 95% CI: −0.06, −0.01) and PS (β = −0.03, 95% CI: −0.04, −0.02) acted as significant negative determinants for the X component. Age and iris center distance showed significant negative determinants in the univariable model, but was not significant in the multivariable model. Other factors, including sex, CCT, CR, ACA, and Iris center Y component, did not show significant effects in univariable GEE.

For the Y component, the iris center Y component was the strongest positive determinant, showing a significant positive association in the multivariable model (β = 0.13, 95% CI: 0.08, 0.18), which indicates that a more centrally positioned iris is independently associated with a higher Y component of angle kappa. Sex also showed a positive correlation in the multivariable model (β = 0.04, 95% CI: 0.02, 0.07). PS showed a negative effect in the multivariable model (β = −0.04, 95% CI: −0.05, −0.03), indicating that larger pupil sizes are independently associated with a decrease in the Y component of angle kappa. CCT had a small negative association in the univariable model (β = −0.0005, 95% CI: −0.001, −0.0001), but this effect was negligible in the multivariable analysis. Other factors, including age, CR, ACD, LT, AL, ACA, WTW, iris center X component, and iris center distance did not show significant effects in the univariable model.

For chord mu, PS and iris center distance showed positive correlations in the multivariable model (β = 0.04, 95% CI: 0.03, 0.05; β = 0.17, 95% CI: 0.11, 0.24, respectively). In contrast, ACD exhibited a negative association in the multivariable model (β = −0.05, 95% CI: −0.08, −0.02). Age, AL, ACA, and iris center X component showed significant effects in the univariable model, but neither was significant in the multivariable model. Other factors including sex, CCT, CR, LT, WTW, and iris center Y component did not show significant effects on chord mu in the univariable model.

## Discussion

This study aimed to evaluate the relationship between ocular biometrics and angle kappa in cataract patients, specifically focusing on the X component, Y component, and chord mu. Using Spearman’s correlation analysis and regression models, we found that PS, ACD, and iris center position were significant determinants of angle kappa. These findings suggest that ocular geometry plays an important role in shaping the optical characteristics of the eye and may influence postoperative outcomes in cataract surgery.

PS showed a significant negative correlation with both the X and Y components of angle kappa, but a positive correlation with chord mu. Specifically, larger pupil sizes were associated with a left shift in angle kappa at the horizontal (X) and an inferior shift in vertical (Y) directions. This relationship suggests that as the pupil expands, it can alter the iris’ position and the anterior segment geometry. On the other hand, larger pupil sizes were positively correlated with chord mu, indicating that as the pupil increases in size, chord mu value also increases. This effect demonstrated the changes in the eye’s geometry that occur with pupil alterations in different lighting conditions (13). Li et al.’s (14) research also showed a positive correlation between larger pupils and chord mu. This effect suggests that changes in pupil size under different lighting conditions cause changes in the angle kappa, possibly due to uneven stress on the iris sphincter. This, in turn, increases the possibility of halos after cataract surgery, and may be one of the reasons for glare after multifocal IOL implantation (14). PS is an important factor to consider during cataract surgery, as it can influence postoperative visual outcomes. According to a previous study, the optimal performance was at sizes 3.0 and 3.5 mm, where the myopic shift was negligible (15). For larger pupil sizes, the far vision peak narrowed, specifically for a pupil diameter of 4.5 mm (16, 17). According to our results, an increase of 1 mm in PS is associated with an increase of 0.04 mm in chord mu. If a patient has a PS of 7 mm, the patient may have a chord mu that is 0.16 mm larger than it is in a patient with a 3 mm PS. A larger chord mu may cause more halos and glare after multifocal IOL implantation. Surgeons should consider each patient’s typical pupil diameter when selecting and calculating the power of the premium IOLs studied, because performance of multifocal IOLs may be reduced if the patient’s pupil is notably smaller or larger than typical during routine activities. Thus, accurate preoperative measurement of PS allows for better predictions of visual disturbances, and can guide the selection of appropriate IOLs, especially in patients requiring toric or multifocal IOLs (18, 19).

ACD showed a positive correlation with the X component of angle kappa, indicating that deeper anterior chambers are associated with left shift of the X components. This is due to the increased distance between the cornea and lens, which alters the angle at which light enters the eye and affects IOL alignment. Conversely, ACD exhibited a negative correlation with chord mu, suggesting that deeper anterior chambers lead to smaller chord mu values, influencing overall refractive characteristics. This means that the geometry of the anterior chamber plays a role in shaping the overall refractive characteristics of the eye, particularly in how light is refracted within the anterior segment. The effect of ACD on angle kappa is particularly important in the context of IOL selection and positioning during cataract surgery. Since angle kappa is a key determinant in IOL alignment, any changes in ACD can affect the IOL’s position relative to the optical axis, potentially leading to postoperative visual distortions such as astigmatism or reduced visual acuity (20). According to the results of our study, an increase of 1 mm in ACD was associated with a decrease of 0.05 mm in chord mu. This means in patients with a shallow ACD, the angle kappa is expected to larger than those with a normal or deep ACD. Thus, IOL selection should consider the effect of ACD on angle kappa. Previous studies have demonstrated that the angle kappa changes before and after cataract surgery (7). This is particularly important in patients with intumescent cataracts, where a shallower preoperative ACD would change to be a much deeper postoperative ACD, resulting in significant reduction of angle kappa. Therefore, in patients with intumescent cataracts, preoperative planning should consider the impact of substantial ACD changes on angle kappa after surgery. Previous studies have shown that a deeper ACD may lead to significant changes in IOL positioning, often requiring adjustments to avoid postoperative refractive errors (20). Specifically, for each 1% increase in ACD, refractive error changes −0.044 D. Thus, a change of 0.179 mm in ACD is required for a 0.25 D variation in refractive error (21). In addition, surgeons should also consider ACD in conjunction with other biometric parameters, such as AL, to better predict the effective power and placement of IOLs, ultimately improving the precision of cataract surgery (22). In summary, ACD significantly affects angle kappa and IOL positioning in cataract surgery, with deeper anterior chambers being associated with smaller chord mu of angle kappa, potentially altering IOL alignment and leading to refractive errors, emphasizing the need to consider ACD along with other biometric parameters for precise IOL selection and optimal refractive outcomes.

The positive correlation between iris center distance and chord mu suggests linkage of the two anatomical landmarks. An increase of 1 mm in iris center distance is associated with an increase of 0.18 mm in chord mu. In Lenstar LS-900 the iris center is considered as the optical center of the eye at the iris level. In patients with high myopia, the fundus center may be distorted (23). Accordingly, the optical center of the anterior segment may also be changed in these patients. Although there is no evidence about the extent of such changes, a 2 mm of alteration in the iris center may cause a 0.35 mm of increase in the chord mu, which is sufficient to exceed known thresholds for perceptible optical effects and cause negative visual quality.

The associations between angle kappa with other ocular biometrics were also investigated in previous studies (24, 25). Our study and these studies have some common determinants of chord mu (ACD and PS), but some determinants in previous research (AL, CR, WTW) were not found significant in our study. The different findings may be due to differences in study populations, measurement devices, and statistical methods. Nevertheless, we have found a new determinant (iris center distance), suggesting that changes in the ocular optical center are also accompanied by changes of the pupil center.

The study provides a detailed investigation into the relationship between ocular biometrics and angle kappa, with a particular focus on the role of PS and ACD in determining angle kappa. The findings provide a detailed analysis of how specific ocular biometric parameters influence the individual components of angle kappa, including the horizontal component (angle kappa X component), the vertical component (angle kappa Y component), and the chord length (chord mu). These results highlight the importance of component-specific preoperative assessment of angle kappa. While existing research has explored the relationship between ocular geometry and angle kappa, this study offers a more nuanced understanding by quantifying the effects of multiple biometric parameters on angle kappa (9, 26). Compared to previous studies, which often examined isolated biometric factors, this research adopts a more comprehensive approach by analyzing the interrelationships among multiple parameters. This systematic framework offers a nuanced understanding of how ocular geometry collectively impacts angle kappa, and thus advances the existing literature by integrating these factors into a unified model. Future studies should validate these relationships in diverse populations and focus on how to refine personalized cataract surgery strategies.

However, this study still has some limitations. Firstly, as a retrospective design, it may be subject to bias and confounding factors, such as variations in measurement techniques or patient selection. Future studies could adopt a prospective design to address these issues. Secondly, the study’s cross-sectional nature does not allow for the assessment of long-term changes in ocular parameters or their impact on postoperative visual outcomes. Longitudinal follow-up studies would provide valuable insight into how these determinants evolve. Thirdly, all subjects were included in the same area, which may have resulted in a selection bias. To uncover more precise results, it is necessary to do further research on a wider range of populations in other places.

## Conclusion

PS and ACD were significant determinants of angle kappa, which provided valuable insights into the role of ocular biometrics in determining angle kappa. Understanding these determinants will give surgeons a better understanding of the relationship between angle kappa and other ocular structures, leading to a more cautious approach in selecting suitable IOL.

## Data Availability

The raw data supporting the conclusions of this article will be made available by the authors, without undue reservation.
